# A randomised control trial of low glycaemic index carbohydrate diet versus no dietary intervention in the prevention of recurrence of macrosomia

**DOI:** 10.1186/1471-2393-10-16

**Published:** 2010-04-23

**Authors:** Jennifer Walsh, Rhona Mahony, Michael Foley, Fionnuala Mc Auliffe

**Affiliations:** 1 Department of Obstetrics and Gynaecology, University College Dublin National Maternity Hospital, Dublin, Ireland

## Abstract

**Background:**

Maternal weight and maternal weight gain during pregnancy exert a significant influence on infant birth weight and the incidence of macrosomia. Fetal macrosomia is associated with an increase in both adverse obstetric and neonatal outcome, and also confers a future risk of childhood obesity. Studies have shown that a low glycaemic diet is associated with lower birth weights, however these studies have been small and not randomised [1,2]. Fetal macrosomia recurs in a second pregnancy in one third of women, and maternal weight influences this recurrence risk [3].

**Methods/Design:**

We propose a randomised control trial of low glycaemic index carbohydrate diet vs. no dietary intervention in the prevention of recurrence of fetal macrosomia.

Secundigravid women whose first baby was macrosomic, defined as a birth weight greater than 4000 g will be recruited at their first antenatal visit.

Patients will be randomised into two arms, a control arm which will receive no dietary intervention and a diet arm which will be commenced on a low glycaemic index diet.

The primary outcome measure will be the mean birth weight centiles and ponderal indices in each group.

**Discussion:**

Altering the source of maternal dietary carbohydrate may prove to be valuable in the management of pregnancies where there has been a history of fetal macrosomia. Fetal macrosomia recurs in a second pregnancy in one third of women. This randomised control trial will investigate whether or not a low glycaemic index diet can affect this recurrence risk.

**Current Controlled Trials Registration Number:**

ISRCTN54392969

## Background

Over one billion adults in the world are now overweight, with more than 300 million clinically obese [4]. In the United States, the prevalence of obesity in women aged 20 - 39 years rose from 9% in 1960 - 1962 to 28% in 1999 - 2000 [4]. In Ireland, mothers are now 10 kg heavier in pregnancy than 20 years ago [5]. Increased maternal body mass index (BMI) confers an elevated risk of delivering a heavier infant[6], while increasing maternal weight gain during pregnancy has been shown to be independently related to increasing infant birth weight[6,7]. The increasing birth weight seen in Ireland in recent decades is a potential cause for concern, not only because of its association with increased instrumental delivery and perineal trauma, but also because of the association between birth weight and childhood obesity.

The westernized diet rich in carbohydrates is thought to contribute to the rates of obesity. Carbohydrate sources are classified according to their induced glucose response which is referred to as glycaemic index [8]. The glycaemic index is defined as the incremental area under the blood glucose curve (AUC) after ingestion of 50 grams of a test food, expressed as a percentage of the AUC of an equal amount of a reference food (usually glucose or white bread)[9]. Low glycaemic index foods induce a low glucose response e.g. whole grain breads, cereals, nuts, dairy foods and high glycaemic index foods induce a high glucose response e.g. processed grains such as flour and bread, snack foods, desserts, soft drinks.

It has been shown that a low glycaemic diet blunts the mid and late pregnancy increase in insulin resistance typically seen in westernized societies [10]. These studies suggest that the loss of insulin sensitivity, which is typical of Western women in the third trimester of pregnancy and is considered to be physiological, may be diet-induced.

Altering the source of maternal dietary carbohydrate may prove to be valuable in the management of pregnancies where there has been a history of fetal macrosomia. Fetal macrosomia recurs in a second pregnancy in one third of women, and maternal weight influences this recurrence risk[3]. We propose to randomise secundigravid women whose first baby was macrosomic (birth weight >4.0 Kg) to receive either no dietary intervention or to be commenced on a low glycaemic index diet during second pregnancy. The primary endpoint of this study is the differences in birth weight between the 2 groups.

## Methods/Design

We will conduct a randomised control trial of low glycaemic index diet versus no intervention in prevention of recurrence of macrosomia.

The primary outcome measure will be the mean birth weight centiles.

The secondary outcome measure will be the difference between the two groups in maternal weight gain in pregnancy.

The study will be carried out in the National Maternity Hospital, Dublin.

Secundigravid women whose first baby was macrosomic, defined as a birth weight greater than 4000 g will be recruited at first booking visit.

Subjects will be excluded if they have diabetes, other medical disorders and those with poor previous pregnancy outcome.

Patients will be randomised into two groups: a control arm which will receive no dietary intervention and a diet arm which will be commenced on a low glycaemic index diet from 14 weeks gestation to delivery under dietetic supervision.

Randomisation will be achieved using computer generated allocations in a ratio of 1:1 contained in sealed opaque envelopes.

Women choosing to enter the study will give written informed consent. Data will be collected on those women approached for study participation and who declined to ensure those participating are representative of this entire group.

Each patient in the diet arm will receive an individualised diet which will be energy matched as appropriate to a patients average caloric intake with the aim of reducing glycaemic load and glycaemic excursions. This diet is eucaloric and is not designed to promote weight loss. In the diet arm each patient will have one dietetic session, in groups of 4-6 at 12-16 weeks' gestation, with the aim of commencing the diet at 16 week's gestation. At booking visit all patients will have their height and weight recorded and their BMI will be calculated (Maternal weight Kg/Height in metres^2^). Additional demographic data including smoking history, SEG and paternal weight and height will be recorded.

In addition to routine care the following additional tests will be performed.

1. Food frequency questionnaire at 12, 28 weeks' gestation and 3 months post partum. Average weekly exercise will also be recorded as part of this questionnaire. The food questionnaire will use questions from section I of the 'Slan national health and lifestyle survey' 2002. This has been validated in an Irish population. At 3 months postpartum the questionnaire will assess compliance to the low glycaemic index diet following pregnancy. Information at 12 weeks will allow baseline information to be obtained. Assessment at 28 week's gestation will assess compliance to the diet in the intervention arm.

2. At each visit maternal weight will be recorded: 12, 20, 28, 34, 36, 38, 40 weeks' gestation.

3. At 12 weeks' fasting blood glucose, mid upper arm circumference and body mass index will be taken.

4. Glucose challenge test at 28 weeks. It is important to determine the presence of gestational diabetes in this group. If gestational diabetes is present care will continue in the multidisciplinary diabetic clinic.

5. Fetal growth ultrasound at 34 weeks. Fetal biometry including anterior abdominal wall thickness will be measured to ensure normal fetal growth velocity and for comparison to the birth weight.

6. Neonatal anthropometry. At delivery, infant birth weight, infant length and head circumference will be recorded in all cases as is current routine practice. The birth weight centile and ponderal index will be computed.

Power analysis was performed by Professor Helen Colhoun. The primary outcome measure is the difference in the birth weight post intervention versus control women, adjusted for birth weight of the first child, i.e. the difference between intervention and control groups in the change in gender and gestational age adjusted birth weight achieved. The sample size required depends on a significance level set at conventional level of 5% and power set at conventional level of 90%. To detect a 0.25 SD difference in birth weight- this is equivalent to a 102 g difference in the birth weights achieved in control and intervention groups one would need 360 women in each group. A sample size of 360 women in each group will have 90% power to detect a difference of at least 100 g in birth weight between control and intervention groups. Thus 720 women will be randomised to 'no intervention' or to 'low glycaemic index diet. Based on 2004 figures at NMH 2700 women annually are secundigravid, with an estimated 20% with a previous baby > 4 kg gives 540 annually available for participation. An estimated 20% (n = 108) will either be ineligible for the study due to inadequate English (14%), history of medical disorder or poor obstetric history (2%), multiple pregnancy (2%), gestational diabetes (2%) leaving 432 women suitable for recruitment. Assuming a 70% participation rate we estimate that up to 300 women annually will be recruited.

This study had received approval from the ethics committee of the National Maternity Hospital (June 2006).

## Discussion

The purpose of this randomised trial is to assess the impact of a maternal eucaloric low glycaemic index diet on birth weight. We will also assess its impact on maternal weight gain in pregnancy. In addition this study will examine the effect of this diet on maternal metabolism, fetal insulin, fetal growth factors and placental development. The results will increase our understanding of the inter-relation between maternal nutrition and pregnancy outcome. If the findings show a beneficial effect of this eucaloric low glycaemic index diet on birth weight and on maternal weight gain, then it may applicable generally to women with increased body mass index, or those at risk of macrosomia. Prevention of macrosomia at birth will help reduce the incidence of childhood and subsequent adult obesity [11]. The outcome of this study will delineate the relationship between maternal nutrition, fetal programming and infant birth weight, and could have a significant impact on the application of public health nutrition policies on maternal and fetal nutrition.

## Competing interests

The authors declare that they have no competing interests.

## Authors' contributions

JW wrote the manuscript, coordinates the day to day running of the study and will analyse and interpret the findings. RM and MF contributed to the design and conduct of the study and contributed to the writing of the manuscript. FMcA conceived and designed the study and contributed to the writing of the manuscript. All authors read and approved the final manuscript.

## Pre-publication history

The pre-publication history for this paper can be accessed here:

http://www.biomedcentral.com/1471-2393/10/16/prepub
